# Supplementary material for: Machine learning accurate exchange and correlation functionals of the electronic density
Source: Nat Commun. 2020 Jul 14;11:3509. doi: 10.1038/s41467-020-17265-7 (PMC7360771; doi:10.1038/s41467-020-17265-7)
Supplement: Supplementary file 1 — Supplementary Information [file 41467_2020_17265_MOESM1_ESM.pdf]

Machine learning accurate exchange and correlation functionals of the electronic density

Dick et al.

Supplementary Note 1 - Related Work

Bogojeski et al. [1] construct a density functional on top of a reasonably cheap baseline DFT calculation (GGA) that can achieve accuracies close to coupled-cluster results. The main difference between our approaches lies in the choice of basis functions, and the way symmetries are encoded. In their method, the molecule is first aligned with a global coordinate system, which is defined through some molecular axes. The electron density is subsequently expanded in a Fourier basis. These design choices seem to restrict their method to systems of fixed size and limit its transferability. Furthermore, instead of obtaining a potential from the energy regressor, an energy correction is added to the baseline results, similar to earlier work by the authors [2]. Thus, to compute forces, their method relies on an auxiliary model that predicts the electron density, whereas we can directly calculate forces using the Hellmann-Feynman [3] theorem.

Nagai et al. [4] propose a more traditional approach of optimizing the functional form of the exchange-correlation (xc) energy. Being defined on a grid without the need for an additional basis set, it is similar in its form to approaches such as DPPS [5], the main difference being the use of a neural network to flexibly parametrize the functional. The neural network is trained by alternating Monte-Carlo updates on the weights with self-consistent calculations, rather than back-propagation. This enables the authors to include densities in their loss-function but limits training set sizes to a few small systems.

Lei and Medford [6] propose a similar, grid-based approach that uses Maxwell-Cartesian spherical harmonic kernels to construct features for their machine-learned functional. However, instead of using total energies as response variables, they rely on a spatial decomposition of the xc-energy, allowing them to decouple grid-points during training but limiting their method to instances for which such decomposition is available.

Apart from approaches rooted in DFT, others have proposed wave-function based methods that try to predict post-Hartree-Fock energies. Welborn et al. [7] and Cheng et al. [8] use molecular-orbital-based machine learning to predict MP2 and coupled-cluster correlation energies with GPR. Nudejima [9] et al. use grid-based descriptors to learn a regression model that can predict the CCSD(T) correlation energy density using densities obtained from Hartree-Fock calculations as input.

To our knowledge, none of the above mentioned methods, except for that of Nagai et al., is used in self-consistent calculations.

Supplementary Note 2 - Datasets

sGDML To test the data efficiency of NeuralXC, we made use of data by Chmiela et al. [10], which was created to evaluate the symmetric gradient-domain machine learning (sGDML) force field model. The dataset contains total energies calculated for benzene, toluene, ethanol, and malonaldehyde at the coupled cluster with singles doubles and perturbative triples (CCSD(T)) level with a cc-pVDZ (cc-pVTZ for ethanol). The calculations were conducted with Psi4 [11]. We further included a set of 1000 water geometries and their associated CCSD(T) total energies calculated with a cc-pVTZ basis set used in Ref. [8] and obtainable at Ref. [12]. The test sets consist of 500 geometries (1000 for ethanol) whereas the maximum training set size was 1000 (500 for water). The reduced training sets were sampled from the full set by employing a k-means clustering algorithm in feature space.

MOB-ML The transferability of our functionals was evaluated by making use of Cheng et al.'s publicly available dataset [12], which contains structures for ethane, propane, n-butane and isobutane sampled from molecular dynamics simulation at 350 K and their associated total energies calculated at a CCSD(T) level (for details see [8]). We further augmented their dataset with our calculations of 100 structures of ethylene, acetylene, and propene each, all sampled from a 5 ps MD trajectory at 350 K and calculated with CCSD(T) using the cc-pVTZ basis [13]. The calculations were conducted with PySCF [14] using density fitting and the frozen core approximation following the methods employed to create the original dataset by Cheng et al.

MB-pol The dataset used to train a functional optimized for water contained 400 water monomers, 500 dimers, and 250 trimers. The structures and their corresponding energies were all sampled from the data that was used to fit the MB-pol force-field [15, 16, 17]. In particular, for dimers and trimers, the sampling consisted of two steps: half the samples were obtained by first binning the structures by their corresponding two and three-body energies and then uniformly sampling from these bins. The other half was obtained by randomly sampling the full datasets. This was done to give more weight to the tails of the data distribution and to capture extreme cases which might contain valuable information for our ML model. As the MB-pol dataset only contained dimers and trimers, we randomly sampled monomers from the dimer structures as well. For the energies, we followed Babin et al. [18] and used the highly accurate Partridge-Schwenke potential energy surface for the monomers and the one-body energies of the dimers [19]. Two and three-body energies were extracted from the MB-pol dataset, where CCSD(T) at the complete basis set (CBS) limit was used to obtain these energies using the MOLPRO [20] package (see Refs. [15, 16] for details).

Supplementary Note 3 - Model transferability

Work on machine learned kinetic energy functionals [21] indicates that the transferability of a given model may depend on its architecture, i.e. the number and size of the hidden layers.

We want to point out that in this work, we have not optimized the architecture or hyperparameters of our neural networks for transferability, rather these hyperparameters were picked to minimize generalization error within the training set, using k-fold cross validation.

For the MOB-ML [8] dataset, we have however optimized the basis set parameters towards better transferability. We have achieved this by choosing a basis that showed the best generalization on a set of 1000 propane structures if the NeuralXC model was trained on 101 methane and 1000 ethane structures. These tests were conducted non-selfconsistently, i.e. trained models were only applied to features (densities) obtained with the baseline method (PBE [22]) and were not used in self-consistent calculations.

We can in principle use the same strategy to determine the optimal network architecture. Supplementary Figure 2 shows the mean absolute error (MAE) on propane of models trained on methane and ethane depending on the number of hidden layers and the number of nodes per hidden layer. For simplicity every hidden layer inside a network has the same number of nodes. We can see that the architecture three hidden layers with 4 nodes each achieve the best transferability. For hidden layers of size 4 and 8, increasing the amount of layers improves transferability up to 3 layers, whereas for a hidden layer of size 16, no obvious trend can be determined. Depending on the network architecture, the MAE of NeuralXC is about 12-22% of the MAE of PBE. For no model architecture NeuralXC produced less accurate energies than PBE.

Supplementary Note 4 - Scaling properties

Neural networks have the convenient property that the cost of evaluating them is independent of the amount of data they were trained on. In contrast, for parameter-free methods such as Kernel Ridge Regression or Gaussian Process Regression the cost of evaluating a model scales with the amount of training data. Nevertheless, our model adds computational overhead to the baseline functional, most of which originates from the projection of the real-space density onto the basis sets. The cost of this projection scales linearly with the number of atoms in the system and we therefore expect for it to be negligible in the limit of large system sizes.

We tested the efficiency of our method by comparing the performance of the NeuralXC functional trained on the MB-Pol dataset to that of PBE and the van-der Waals functional with consistent exchange (vdW-cx) [23]. The started out with a system containing 12 water molecules inside a 6.51Å× 6.51Å× 6.76Å tetragonal unit cell and proceeded by repeatedly doubling the unit cell to obtain larger systems. All systems were calculated with a doubly polarized quadruple zeta

basis [24] and a real-space cutoff of 300 Ry. The calculations were run with SIESTA [25]. We have opted to simulate short molecular dynamics (MD) trajectories of 10 steps, and report CPU hours per MD step, in order to approximately disregard start-up times.

For all functionals, the scaling is approximately linear for small systems as contributions from real-space grid calculations dominate the computational workload. NeuralXC is approximately 14% more expensive than PBE and 30% cheaper than vdW-cx for a system containing 12 water molecules. The gap between all three methods decreases once system sizes reach a domain where the cubic scaling property of the diagonalization algorithm is starting to dominate the workload.

Supplementary Note 5 - Intra-group transferability

Beyond the studies presented in the main text, we have also tested how well our method generalizes to elements other than those contained in the training set. It is reasonable to assume that a model should be transferable within the same group of the periodic table due to the similar chemistry involved. We do, however, not expect to see transferability within rows.

We have trained a model on an extensive dataset containing samples of all molecules studied in the main text. The data set used for pre-training consisted of 50 toluene, 20 benzene, 250 malonaldehyde, 100 ethanol, 50 water, 50 ethane, 20 propane, 10 ethylene and 10 acetylene structures calculated with the density functional ωB97M-V and the 6-311G* basis set using PySCF.

A model with three hidden layers and four nodes per hidden layer was trained on this dataset. The layers were frozen and a second model was trained on a "fine-tuning" data set of 50 water, 50 ethane, 10 ethylene, 10 acetylene calculated with CCSD(T) using a cc-pVQZ basis set. The second model also consisted of three hidden layers with 4 nodes each and was trained as an additive correction to the first model. The basis set parameters used are the same as for the MOB-ML model, given in Supplementary Table 1.

As we were aiming to create a functional that can be used across a wide variety of elements we have not encoded any information about the atomic species in the model input. In other words, we have used the same basis set, and the same atomic neural network for every element in the training set. This is in contrast to other parts of this work, where a different atomic network was used for every species.

Supplementary Table 2 shows that our model improves bond lengths overall. Being trained on systems containing Oxygen and Carbon, it seems to be transferable to Silicon and Sulfur. No significant improvement in bond angles can be observed.

We want to point out that these results should be considered a proof of concept and not as an attempt to obtain a general-purpose functional. While the latter goal is definitely worth pursuing it will require a more curated training set than the one used in this study. This avenue will be explored in future work.

Supplementary Note 6 - s66 dataset

The s66x8 dataset [26], which contains dissociation curves for 66 non-covalent complexes relevant to biomolecular structures, was used to evaluate the transferability of our water model to heterogeneous systems. The reference energies and structures were taken from Ref. [26]. Energies were obtained at the CCSD(T) level of theory, structures were obtained with MP2/cc-pVTZ using the MOLPRO package. For further details regarding the calculations we refer the reader to ref. [26].

We evaluated the performance of NXC-W01 by optimizing the intermolecular distances in the complexes while holding intramolecular distances fixed. The same was done using PBE to compare our machine learned model to its baseline functional.

Supplementary Figure 3 and Supplementary Table 3 report the absolute errors in equilibrium distance with respect to the reference data . We have only reported results for the hydrogen-bonded subset of s66. Other complexes (dispersion and "other") did not improve significantly. This is expected as the model was not fitted to treat the latter interactions. For hydrogen-bonded

system, we observe that NXC-W01 improves bonding distances from an average error of 0.038 Å for PBE to an error of 0.020 Å.

Beyond bond lengths, we have also examined how well NeuralXC reproduces binding energies across the eight intermolecular separations included for every molecule pair in the s66x8 dataset. We report average RMS errors for these energies. While these two quantities are of course closely related, the bond length places more importance on an accurate treatment of energies close to the equilibrium distance, whereas an energy metric gives the same weight to all distances in the s66x8 dataset (these range from 0.9 to 2.0 times the equilibrium distance).

For hydrogen bonded systems NXC produces an RMSE of 7.2 meV compared to an error of 9.9 meV for PBE. The largest improvement can be observed for water...pyridine where NXC produces an average error of 4.8 meV and PBE an error of 12.7 meV. For water..methanol NXC shows the largest decline in performance compared to PBE with an error of 5.3 meV and 2.4 meV respectively.

For dispersion dominated systems NXC produces an RMSE of 53.7 for NXC and 53.8 for PBE, with the largest improvement occuring for uracil...uracil (78.2 meV vs. 87.3 meV) and the largest decline for benzene...benzene (59.5 meV vs. 58.0 meV)

For systems classified as "other" in the original dataset NXC produces an RMSE of 23.5 meV compared to 24.2 meV for PBE. The largest improvement can be observed for benzene...acetamide (14.6 meV vs 18.6 meV) the largest decline in accuracy for ethyne...water(3.5 meV vs 1.2 meV)

To summarize, while hydrogen bond lengths are corrected by a factor of about 50%, we observe no significant improvement in the overall energetic treatment of systems contained in the s66x8 dataset. We are, however, encouraged by our findings that NeuralXC, when used beyond the scope of its training set, does not decrease the accuracy of its baseline functional PBE. For future applications of NXC-W01 this means that the functional can be used in a variety of water-containing systems such as solvated molecules, where a very accurate treatment of water-water interactions can be achieved while a PBE-level treatment of the remaining interactions is obtained.

Supplementary Note 7 - Fitting to the exact potential

Our results indicate that while fitting a model to the total energy certainly improves the prediction of energies and forces it does little to improve electron densities and quasi particle energies. Hence, we will outline a path to solve this problem by fitting to the exact potential instead, and will show preliminary results that will give an outlook on further studies regarding this issue.

Assuming the exact potential V_{ref} for a given system is known, we can use it to define an extended loss function. We use the term "exact" in the sense that it reproduces the reference ground state density ρ_{ref} associated with the reference energy E_{ref}.

We define a new loss-function as an extension to Supplementary Equation (7):

$$\mathcal{L} = \mathcal{L}_{\mathrm{E}} + \lambda^2 \mathcal{L}_{\mathrm{V}} = \sum_i^N ((E_{\mathrm{ref}}^{(i)} - E_0^{(i)}) - E_{\mathrm{ML}}[\rho_{\mathrm{ref}}^{(i)}])^2 + \lambda^2 \sum_i^N \int_{\mathbf{r}} ((V_{\mathrm{ref}}^{(i)}(\mathbf{r}) - V_0^{(i)}(\mathbf{r})) - V_{\mathrm{ML}}[\rho_{\mathrm{ref}}^{(i)}(\mathbf{r})])^2 \quad (1)$$

with

$$V_{\mathrm{ML}}[\rho(\mathbf{r})] = \sum_\beta \frac{\partial E_{\mathrm{ML}}}{\partial c_\beta} \psi_\beta(\mathbf{r}) \equiv \sum_\beta v_\beta \psi_\beta(\mathbf{r}) \quad (2)$$

$$V_0 = \frac{\delta E_0}{\delta n} \quad (3)$$

. The scale factor λ can be chosen to give different priorities to the energy and potential parts of the loss function and it might be beneficial to adjust it during the training process. Expanding V_{ref} in the machine-learning basis

$$V_{\mathrm{ref}}(\mathbf{r}) = V_0 + \sum_\beta b_\beta \psi_\beta(\mathbf{r}), \quad (4)$$

the loss function simplifies to

$$\mathcal{L} = \mathcal{L}_{\mathrm{E}} + \lambda^2 \sum_i^N \sum_{\alpha\beta} (v_\alpha S_{\alpha\beta} v_\beta - 2 v_\alpha S_{\alpha\beta} b_\beta). \tag{5}$$

where $S_{\alpha\beta} = \int_{\mathbf{r}} \psi_\alpha(\mathbf{r})\psi_\beta(\mathbf{r})$. We optimized a model on a set of five H_2 molecules (see Supplementary Figure 5) with bond lengths between 0.55 and 0.85 Å that were computed using an aug-cc-pVQZ basis with CCSD. The effective one-body potential V_{ref} was calculated using our implementation of a method developed by Wu et al. [27] together with data created with PySCF . For the machine learning basis set ψ, we have also used aug-cc-pVQZ to simplify the computation of integrals involving atomic orbitals.

To evaluate the quality of the obtained density we use the following error metric:

$$\mathrm{Error}_{\rho_{\mathrm{xc}}} = \frac{\int_r |\rho_{\mathrm{xc}} - \rho_{\mathrm{ref}}|^2}{\int_r |\rho_{\mathrm{xc}}|^2 \int_r |\rho_{\mathrm{ref}}|^2} \tag{6}$$

which evaluates to a value between zero and one, where zero indicates perfect agreement. The same metric was used in work by Zhou et al. [28]. Both Supplementary Figures 4 and 5 indicate that there is signifcant improvement over the baseline functional PBE.

Further investigation of this method and an integration of the extended loss in the official release of NeuralXC will be the subject of future works.

Supplementary Methods

In Supplementary Table 1, we summarized the basis sets used for every ML-model, their number of radial functions n_{max}, their highest angular momentum l_{max} and their radial cutoff r_{o} as well as the network architecture (meaning the number of hidden layers and the amount of nodes per layer). The "Density" column indicates whether we used the full density (ρ) or the modified density ($\delta\rho$).

We have already discussed the "iterative training" procedure in the Methods section of the main text, but we will outline it here in more detail for clarity:

Given a set of structures $\mathcal{C} = \{\mathbf{R}_j^{(i)}, Z_j^{(i)}\}$ ($i \in \{1, ..., N\}$ and N = number of training samples) and corresponding total energies $\{E_{\mathrm{ref}}^{(i)}\}$ obtained with a reference method of choice (e.g. coupled-cluster), we pick a baseline density functional $E_{\mathrm{base}}[\rho] \equiv E_0[\rho]$ (PBE in this work). We use the baseline functional to obtain self-consistent ground state densities and total energies for $\mathcal{C}$ which we denote by $\rho_0^{(i)}$ and $E_0^{(i)}$. We introduce the (first iteration) machine learned functional $E_{\mathrm{ML},1}[\rho; \omega, \theta]$ that consists of a fully connected neural network plus preprocessing steps as described in the Methods section of the main text. Here ω denotes the model parameters inside the neural network, whereas θ denotes the hyperparameters of the model.

Given a choice of hyperparameters θ, the model parameters ω can be optimized by minimizing the loss function

$$\mathcal{L}_{\mathrm{train},\rho_0} = \sum_i^N ((E_{\mathrm{ref}}^{(i)} - E_0^{(i)}) - E_{\mathrm{ML},1}[\rho_0^{(i)}; \omega, \theta])^2, \tag{7}$$

using gradient descent. We will denote the optimized model parameters as $\hat{\omega}$.

The hyperparameters are then optimized using k-fold cross-validation. This means the above procedure was repeated for different choices of hyperparameters (performing a grid search). For each choice of θ, the training set was split into K random folds $\mathcal{C}_k$ (with $k \in \{1, 2, ..., K\}$). Let $S_k(\theta)$ be the score assigned to fold $\mathcal{C}_k$, if the model was fitted on data contained in all other folds $\mathcal{C}_{j \neq k}$ with hyperparameters θ:

$$S_k(\theta) = \frac{1}{|C_k|} \sum_{i \in \mathcal{C}_k} |(E_{\mathrm{ref}}^{(i)} - E_0^{(i)}) - E_{\mathrm{ML},1}[\rho_0^{(i)}; \hat{\omega}, \theta]|. \tag{8}$$

Then the optimized hyperparameters $\hat{\theta}$ are given by

$$\hat{\theta} = \underset{\theta}{\operatorname{argmin}} S(\theta) = \frac{1}{K} \sum_{k=1}^{K} S_k(\theta). \tag{9}$$

We have now obtained an optimized functional $E_1[\rho] = E_0[\rho] + E_{\mathrm{ML},1}[\rho; \hat{\omega}, \hat{\theta}]$. If we were to only use the machine learned model as an additive energy correction, the model could be used as is. In self-consistent calculations, however, using the modified functional $E_1[\rho]$ will lead to new ground state densities $\rho_1^{(i)} \neq \rho_0^{(i)}$. Therefore the "self-consistent loss" $\mathcal{L}_{\mathrm{SC}}$ will generally be greater than the training loss $\mathcal{L}_{\mathrm{train}}$:

$$\mathcal{L}_{\mathrm{SC},1} = \sum_{i}^{N} ((E_{\mathrm{ref}}^{(i)} - E_0[\rho_1^{(i)}]) - E_{\mathrm{ML},1}[\rho_1^{(i)}; \hat{\omega}, \hat{\theta}])^2 \geq \mathcal{L}_{\mathrm{train},\rho_0}. \tag{10}$$

To remedy this, we can apply the above optimization procedure iteratively, introducing a new machine learned functional $E_{\mathrm{ML},2}$ that is trained to minimize

$$\mathcal{L}_{\mathrm{train},\rho_1} = \sum_{i}^{N} ((E_{\mathrm{ref}}^{(i)} - E_0[\rho_1^{(i)}]) - E_{\mathrm{ML},2}[\rho_1^{(i)}; \omega, \theta])^2, \tag{11}$$

obtaining new self-consistent densities $\rho_2^{(i)}$ and so on, until convergence with respect to an energy tolerance $\epsilon = 0.5$ meV is achieved, i.e.

$$|\mathcal{L}_{\mathrm{SC},j} - \mathcal{L}_{\mathrm{train},\rho_{j-1}}| < \epsilon^2. \tag{12}$$

Note that in order for this iterative training to converge, it is advisable to have a clearly defined relationship between $E_{\mathrm{ML},j}$ and $E_{\mathrm{ML},j-1}$. One option is to simply take $E_{\mathrm{ML},j} = E_{\mathrm{ML},j-1}$ and continue the optimization of the functional starting with the parameters $\hat{\omega}_{j-1}$ obtained during the previous iteration. We have found the following procedure to give the best convergence: We obtain $E_{\mathrm{ML},j}$ by freezing all hidden layers of $E_{\mathrm{ML},j-1}$ and concatenating new hidden layers to it. The parameters associated with the new hidden layers are then to be optimized in order to minimize $\mathcal{L}_{\mathrm{train},\rho_{j-1}}$. Cross-validation, as described above, can still be applied at every iterative training step to determine the optimal number of nodes and hidden layers, however hyperparameters that are associated with the preprocessing pipeline remain fixed after the first iteration.

Once this iterative training procedure has converged after J steps, we can freeze the model $E_{\mathrm{ML},J}[\rho]$ and use $E_{\mathrm{NXC}} = E_{\mathrm{base}}[\rho] + E_{\mathrm{ML},J}[\rho]$ as a functional. In this work, we have opted to only add one layer per iterative step, therefore, the number of iterations J is equivalent to the number of hidden layers in the finished network. Supplementary Table 1 gives an overview of the model architectures: e.g. [4, 4, 4] indicates a fully connected neural network consisting of 3 hidden layers with 4 nodes each. We can therefore conclude that the training converged after 3 iterations in this case.

In many cases, we were able to improve accuracy even further, by using E_{NXC} as the new baseline functional and restarting the entire training procedure. Doing so, one obtains an ensemble of functionals. In Supplementary Table 1 we have indicated whenever an ensemble of functionals was employed, by the use of a plus sign in the "network architecture" column. It should be noted that in spite of the terminology used here, our method differs from traditional ensemble learning by the fact that every part of the model 'sees' the entire training set. Ensemble methods such as bootstrap aggregating ("bagging") [29] only use subsets of the training data to train a single predictor in order to enhance generalization. In our case, using ensembles merely enhances the expressivity of the overall model.

While the training procedure described above might seem slightly involved, we have automatized everything in the NeuralXC implementation so that the end-user can train a functional by using a single command.

1 Supplementary Figures

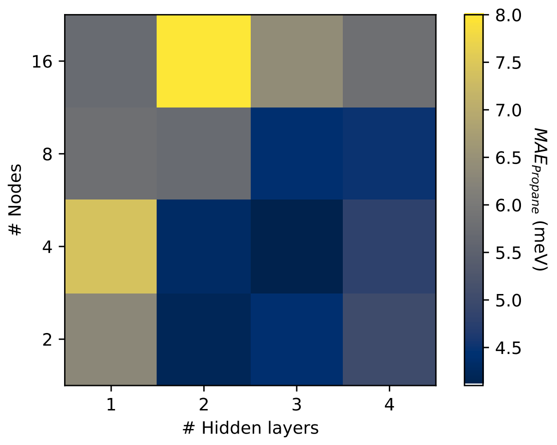

Supplementary Figure 1: Mean absolute error (MAE) of a NeuralXC model trained on 100 structures of methane and 1000 structures of ethane applied to 1000 structures of propane (data taken from MOB-ML dataset [8]) depending on the number of hidden layers and nodes. The MAE of PBE for the same systems (i.e. the baseline error) is 35 meV.

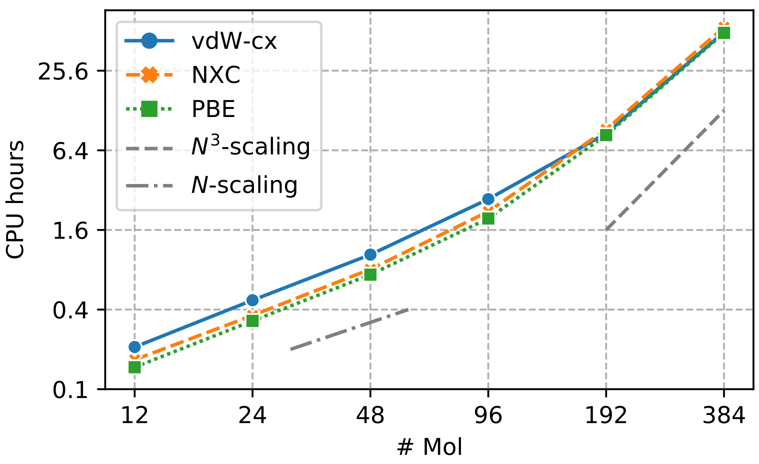

Supplementary Figure 2: CPU hours per MD step with respect to system size and XC-functional used. Note that both axes use a logarithmic scale.

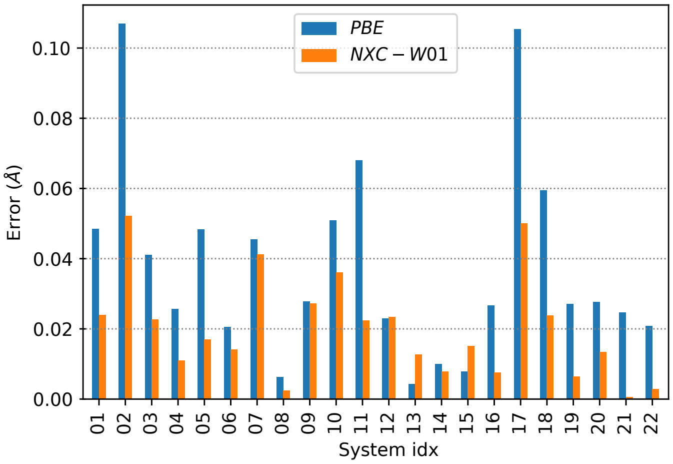

Supplementary Figure 3: Absolute errors in equilibrium intermolecular distances for all hydrogen-bonded systems in the s66 dataset. Compared are PBE and NXC-W01, the NeuralXC model trained on the MB-Pol dataset. For molecule names, please refer to Supplementary Table 3

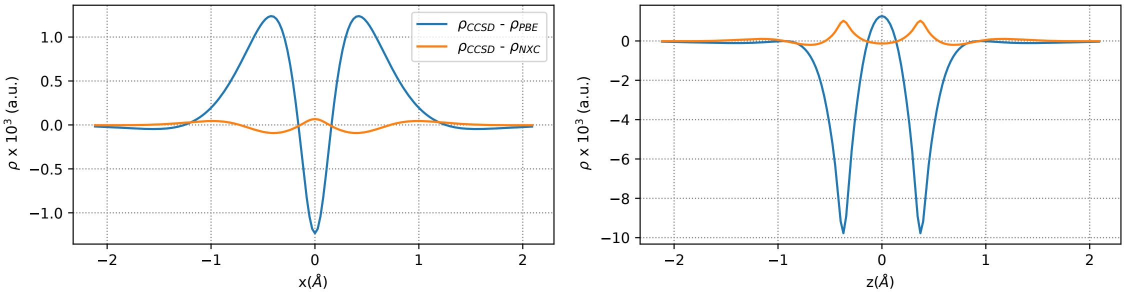

Supplementary Figure 4: One dimensional cuts of the electron density. (left) Electron density on an axis orthogonal to the bonding axis, with the Hydrogen atom positioned at $x = 0$. (right) Electron density along the bonding axis. Both plots were computed for an H_2 molecule at experimental equilibrium geometry ($d = 0.74$ Å), a configuration that was not contained in the training set.

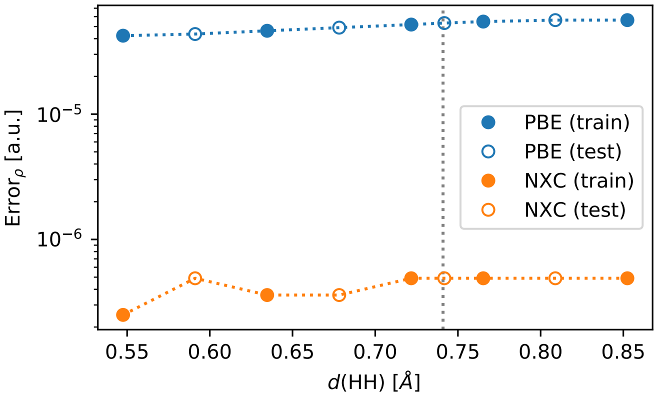

Supplementary Figure 5: Error metric for PBE and NeuralXC for all geometries contained in training (solid circles) and test (empty circles) set. The vertical gray line indicates the experimental equilibrium geometry used in Supplementary Figure 4.

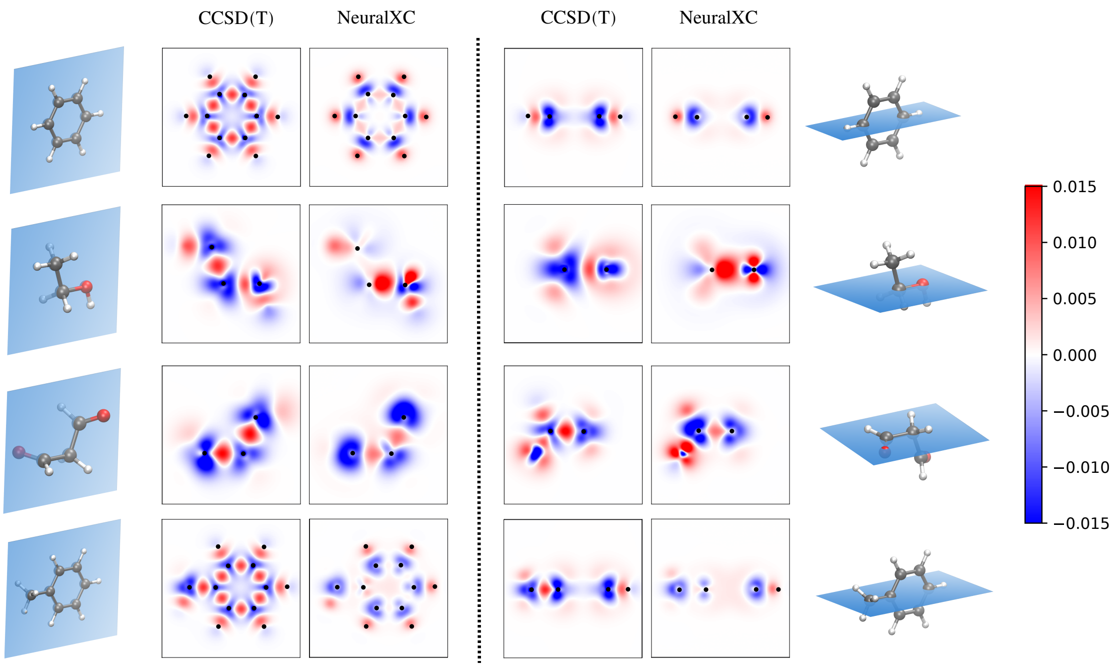

Supplementary Figure 6: Comparison of the difference in electron density between CCSD(T) and PBE and NeuralXC and PBE for benzene, ethanol, malonaldehyde and toluene. Two dimensional cuts either correspond to high-symmetry planes or planes containing a significant number of atoms and are indicated by blue surfaces in the molecule depictions adjacent to the density plots. Black dots inside the density plots indicate the positions of in-plane atoms. Atoms are color-coded with red corresponding to oxygen, white to hydrogen and grey to carbon. NeuralXC models correspond to the ones presented in the "Data-efficiency" section of the main text.

2 Supplementary Tables

Model/Dataset	Density	$n_{\max}$	$l_{\max}$	r_{o}[Å]	Network architecture
sGDML(Benzene)	$\delta\rho$	4	4	2	[4, 4] + [4]
sGDML(Toluene)	$\delta\rho$	4	4	2.5	[8, 8] + [8]
sGDML(Ethanol)	$\delta\rho$	4	4	2.5	[8, 4, 8] + [4, 8] + [4]
sGDML(Malonaldehyde)	$\delta\rho$	4	4	2.5	[4, 8, 8] + [4, 8]+ [4] + [4]
sGDML(Water)	$\delta\rho$	4	3	1.5	[4, 4, 4] + [4, 4]
MOB-ML	$\delta\rho$	5	4	2.5	[4, 4, 4]
NXC-W01	ρ	4	4	2	
(1&2-body)					[4, 4] + [4] + [4]
(3-body)					[4]

Supplementary Table 1: Basis set parameters for the number of radial functions $n_{\max}$, the maximum angular momentum $l_{\max}$, the cutoff radius r_{o} and the kind of density used ($\delta\rho$ or ρ). The network architecture should be interpreted as follows: A fully connected neural network (FCNN) is indicated by square brackets, inside, the number of nodes per hidden layer is specified. For example, [4,4,4] corresponds to a FCNN with three hidden layers with four nodes each. If the total network consists of a sum of multiple FCNN, this is signaled by a plus sign between the networks. The overall architecture is not chosen a-priori but determined by cross-validation (for the number of nodes) and the convergence of the iterative training procedure (for the number of layers per FCNN).

			Exp.	PBE	NXC	Δ PBE	Δ NXC
SiH_4 [30]	dist.	SiH	1.480	1.504	1.487	0.024	0.008
SiO [31]	dist.	SiO	1.510	1.536	1.526	0.026	0.016
SH [32]	dist.	SH	1.341	1.365	1.356	0.024	0.015
SH_2 [33]	dist.	SH	1.336	1.360	1.352	0.025	0.017
	θ	HSH	92.1	91.2	91.3	0.9	0.8
S_2H_2 [34]	dist.	SS	2.056	2.065	2.063	0.009	0.005
		SH	1.342	1.373	1.362	0.031	0.020
	θ	SSH	97.9	98.7	98.0	0.8	0.1
SOH_2 [35]	dist.	SO	1.662	1.691	1.689	0.029	0.027
		SH	1.342	1.378	1.363	0.036	0.021
		OH	0.961	0.970	0.960	0.009	0.001
	θ	OSH	98.6	98.8	97.6	0.2	0.9
		SOH	107.2	106.7	107.1	0.5	0.1
Mean d						0.024(3)	0.014(3)
Mean θ						0.6(2)	0.5(2)

Supplementary Table 2: Bond lengths and angles for the equilibrium geometries obtained with PBE, NXC and compared to experimental results. Distances are given in Å, bonda angles θ are given in degrees.

	System	PBE	NXC-W01	MP2 (ref)	ΔPBE	ΔNXC-W01
01	Water ... MeOH	1.914	1.938	1.962	0.049	0.024
02	Water ... $MeNH_2$	1.905	1.960	2.012	0.107	0.052
03	Water ... Peptide	1.864	1.882	1.905	0.041	0.023
04	MeOH ... MeOH	1.911	1.926	1.937	0.026	0.011
05	MeOH ... $MeNH_2$	1.921	1.952	1.969	0.048	0.017
06	MeOH ... Peptide	1.861	1.867	1.881	0.021	0.014
07	MeOH ... Water	1.940	1.944	1.985	0.046	0.041
08	$MeNH_2$ ... MeOH	2.269	2.265	2.263	0.006	0.002
09	$MeNH_2$ ... $MeNH_2$	2.304	2.304	2.277	0.028	0.027
10	$MeNH_2$ ... Peptide	2.292	2.277	2.241	0.051	0.036
11	$MeNH_2$ ... Water	1.912	1.957	1.980	0.068	0.022
12	Peptide ... MeOH	2.038	2.038	2.015	0.023	0.023
13	Peptide ... $MeNH_2$	2.061	2.078	2.066	0.004	0.013
14	Peptide ... Peptide	3.292	3.289	3.282	0.010	0.008
15	Peptide ... Water	2.073	2.066	2.081	0.008	0.015
16	Uracil ... Uracil (BP)	1.807	1.826	1.833	0.027	0.008
17	Water ... Pyridine	1.890	1.945	1.996	0.105	0.050
18	MeOH ... Pyridine	1.893	1.929	1.953	0.059	0.024
19	AcOH ... AcOH	1.664	1.698	1.692	0.027	0.006
20	$AcNH_2$ ... $AcNH_2$	1.831	1.845	1.859	0.028	0.013
21	AcOH ... Uracil	1.679	1.704	1.704	0.025	0.001
22	$AcNH_2$ ... Uracil	1.868	1.886	1.889	0.021	0.003
Mean					0.038(6)	0.020 (3)

Supplementary Table 3: Equilibrium intermolecular distances for all hydrogen-bonded systems in the s66 dataset calculated with PBE, NXC-W01 and MP2 (with cc-pVTZ basis; reference data s66). Errors w.r.t. MP2 are given in the columns starting with Δ. We report the mean error and its standard error in the last row.

Supplementary References

[1] Bogojeski, M., Vogt-Maranto, L., Tuckerman, M. E., Mueller, K.-R. & Burke, K. Density functionals with quantum chemical accuracy: From machine learning to molecular dynamics, preprint at 10.26434/chemrxiv.8079917.v1 (2019).

[2] Dick, S. & Fernandez-Serra, M. Learning from the density to correct total energy and forces in first principle simulations. *J. Chem. Phys.* **151**, 144102 (2019).

[3] Feynman, R. P. Forces in molecules. *Phys. Rev.* **56**, 340 (1939).

[4] Nagai, R., Akashi, R. & Sugino, O. Completing density functional theory by machine learning hidden messages from molecules. *Npj Comput. Mater.* **6**, 1–8 (2020).

[5] Fritz, M., Fernández-Serra, M. & Soler, J. M. Optimization of an exchange-correlation density functional for water. *J. Chem. Phys.* **144**, 224101 (2016).

[6] Lei, X. & Medford, A. J. Design and analysis of machine learning exchange-correlation functionals via rotationally invariant convolutional descriptors. *Phys. Rev. Mater.* **3**, 063801 (2019).

[7] Welborn, M., Cheng, L. & Miller III, T. F. Transferability in machine learning for electronic structure via the molecular orbital basis. *J. Chem. Theory Comput.* **14**, 4772–4779 (2018).

[8] Cheng, L., Welborn, M., Christensen, A. S. & Miller III, T. F. A universal density matrix functional from molecular orbital-based machine learning: Transferability across organic molecules. *J. Chem. Phys.* **150**, 131103 (2019).

[9] Nudejima, T., Ikabata, Y., Seino, J., Yoshikawa, T. & Nakai, H. Machine-learned electron correlation model based on correlation energy density at complete basis set limit. *J. Chem. Phys* **151**, 024104 (2019).

[10] Chmiela, S., Sauceda, H. E., Müller, K.-R. & Tkatchenko, A. Towards exact molecular dynamics simulations with machine-learned force fields. *Nat. commun.* **9**, 3887 (2018).

[11] Parrish, R. M. *et al.* Psi4 1.1: An open-source electronic structure program emphasizing automation, advanced libraries, and interoperability. *J. Chem. Theory Comput.* **13**, 3185–3197 (2017).

[12] Cheng, L., Welborn, M., Christensen, A. S. & Miller, T. F. Thermalized (350k) qm7b, gdb-13, water, and short alkane quantum chemistry dataset including mob-ml features (2019). URL `https://data.caltech.edu/records/1177`.

[13] Dunning Jr, T. H. Gaussian basis sets for use in correlated molecular calculations. i. the atoms boron through neon and hydrogen. *J. Chem. Phys.* **90**, 1007–1023 (1989).

[14] Sun, Q. *et al.* Pyscf: the python-based simulations of chemistry framework (2017). URL `https://onlinelibrary.wiley.com/doi/abs/10.1002/wcms.1340`.

[15] Babin, V., Leforestier, C. & Paesani, F. Development of a "first principles" water potential with flexible monomers: Dimer potential energy surface, VRT spectrum, and second virial coefficient. *J. Chem. Theory Comput.* **9**, 5395–5403 (2013).

[16] Babin, V., Medders, G. R. & Paesani, F. Development of a "first principles" water potential with flexible monomers. ii: Trimer potential energy surface, third virial coefficient, and small clusters. *J. Chem. Theory Comput.* **10**, 1599–1607 (2014).

[17] Medders, G. R., Babin, V. & Paesani, F. Development of a "first-principles" water potential with flexible monomers. iii. liquid phase properties. *J. Chem. Theory Comput.* **10**, 2906–2910 (2014).

[18] Bartók, A. P., Kondor, R. & Csányi, G. On representing chemical environments. *Phys. Rev. B* **87**, 184115 (2013).

[19] Partridge, H. & Schwenke, D. W. The determination of an accurate isotope dependent potential energy surface for water from extensive ab initio calculations and experimental data. *J. Chem. Phys.* **106**, 4618–4639 (1997).

[20] Werner, H.-J., Knowles, P. J., Knizia, G., Manby, F. R. & Schütz, M. Molpro: a general-purpose quantum chemistry program package. *Wiley Interdiscip. Rev. Comput. Mol. Sci.* **2**, 242–253 (2012).

[21] Golub, P. & Manzhos, S. Kinetic energy densities based on the fourth order gradient expansion: performance in different classes of materials and improvement via machine learning. *Phys. Chem. Chem. Phys.* **21**, 378–395 (2019).

[22] Perdew, J. P., Burke, K. & Ernzerhof, M. Generalized gradient approximation made simple. *Phys. Rev. Lett.* **77**, 3865 (1996).

[23] Berland, K. & Hyldgaard, P. Exchange functional that tests the robustness of the plasmon description of the van der waals density functional. *Phys. Rev. B* **89**, 035412 (2014).

[24] Corsetti, F., Fernández-Serra, M., Soler, J. M. & Artacho, E. Optimal finite-range atomic basis sets for liquid water and ice. *J. Phys. Condens. Matter* **25**, 435504 (2013).

[25] Soler, J. M. *et al.* The siesta method for ab initio order-n materials simulation. *J. Phys. Condens. Matter* **14**, 2745 (2002).

[26] Rezác, J., Riley, K. E. & Hobza, P. S66: A well-balanced database of benchmark interaction energies relevant to biomolecular structures. *J. Chem. Theory Comput.* **7**, 2427–2438 (2011).

[27] Wu, Q. & Yang, W. A direct optimization method for calculating density functionals and exchange–correlation potentials from electron densities. *J. Chem. Phys.* **118**, 2498–2509 (2003).

[28] Zhou, Y., Wu, J., Chen, S. & Chen, G. Toward the exact exchange–correlation potential: A three-dimensional convolutional neural network construct. *J. Phys. Chem. Lett.* **10**, 7264–7269 (2019).

[29] Breiman, L. Bagging predictors. *Machine learning* **24**, 123–140 (1996).

[30] Boyd, D. Infrared spectrum of trideuterosilane and the structure of the silane molecule. *The Journal of Chemical Physics* **23**, 922–926 (1955).

[31] Kohn, W. & Sham, L. J. Diatomic spectral database. *NIST Standard Reference Database* **114** (2005).

[32] Huber, K.-P. *Molecular spectra and molecular structure: IV. Constants of diatomic molecules* (Springer Science & Business Media, 2013).

[33] Cook, R. L., De Lucia, F. C. & Helminger, P. Molecular force field and structure of hydrogen sulfide: Recent microwave results. *J. Mol. Struct.* **28**, 237–246 (1975).

[34] Behrend, J., Mittler, P., Winnewisser, G. & Yamada, K. Spectra of deuterated disulfane and spectroscopic determination of its molecular structure. *J. Mol. Spectrosc.* **150**, 99–119 (1991).

[35] Baum, O. *et al.* Gas-phase detection of hsod and empirical equilibrium structure of oxadisulfane. *J. Mol. Struct.* **795**, 256–262 (2006).
